# False‐positive test results in diagnosing allergy to glatiramer acetate: Case report and a systematic literature review

**DOI:** 10.1002/iid3.357

**Published:** 2020-10-05

**Authors:** Stefani Röseler, Friederike Leufgens, Hans F. Merk, Jens M. Baron, Silke Moll‐Slodowy, Gerda Wurpts, Galina Balakirski

**Affiliations:** ^1^ Department of Pneumology, Allergology, Sleep and Respiratory Medicine Augustinians Hospital Cologne Germany; ^2^ Department of Dermatology and Allergology University Hospital of RWTH Aachen Aachen Germany; ^3^ Clinic for Hematology, General Oncology and Palliative Care Hospital Düren Düren Germany; ^4^ Department of Dermatology and Allergology University Hospital of Bonn Bonn Germany

Glatiramer acetate (GA; trade name: Copaxone®) is a mixture of synthetic polypeptides that is used for the immunomodulatory treatment of relapsing‐remitting multiple sclerosis (RRMS).^1^ While some mechanisms of action of GA are still not completely understood, it has been shown to bind to major histocompatibility complex molecules and to induce an in vivo change of the cytokine secretion pattern of CD4+ and CD8+ T cells.^1^

Injection site reactions and immediate post‐injection systemic reactions (IPISR) are adverse events caused by GA that are most frequently described in the literature.^2^ The latter are characterized by flushing, chest tightness, palpitations, and shortness of breath, and may occur several minutes after the injection, but resolve spontaneously, usually within 1 h. IPISR can take the form of a single episode or recurrent event. The mechanism of this reaction is still unclear.^2^ Moreover, as the use of GA became more common in clinical practice, a number of reports on possible type I hypersensitivity reactions to this drug were released.^3^, ^4^, ^5^, ^6^, ^7^, ^8^, ^9^, ^10^, ^11^, ^12^, ^13^ In most cases, a positive skin prick test, an intradermal test, or the basophil activation test (BAT) led to the diagnosis of an immediate‐type hypersensitivity reaction to GA and to a subsequent cessation of the therapy.^5^, ^7^, ^8^, ^12^, ^13^

At the present time, there are no generally accepted recommendations on how allergy diagnostic testing should be carried out to evaluate these reactions to GA. Skin prick and intradermal tests are performed at dilution ratios of 1:10000, 1:1000, 1:100, and sometimes 1:10 or with the undiluted substance.^3^, ^5^, ^7^, ^9^, ^11^, ^12^, ^13^ However, this procedure is not validated or standardized with respect to irritation or false‐positive reactions.

Herein we report a case of a 38‐year‐old man who was examined at our allergology clinic due to complaints of intermittent generalized pruritic skin lesions. Having received the diagnosis of RRMS in 2009, he was prescribed 20 mg GA per day (changed to 20 mg three times per week starting from 2015). From the onset of the immunomodulatory therapy, he regularly developed itchy erythematous skin reactions at the injection site and, from time to time, solitary skin lesions, which could clearly be identified as hives in the photos taken by the patient. Starting from 2016, an almost daily appearance of hives could be observed, regardless of the time of the GA injections. In addition, angioedema of the lips, tongue, and genital area occurred twice. Since cutaneous reactions have been described as possible side effects of GA, the therapy was discontinued. Despite the discontinuation of GA, the symptoms persisted with undiminished intensity. The patient presented to our allergological department approximately 18 months after the onset of the symptoms (and 2 months after the discontinuation of GA). The patient denied any known allergies. Apart from the RRMS, his medical history was remarkable for hypothyroidism, which was being treated with l‐thyroxin 75 µg once daily.

After obtaining informed consent from the patient, we performed a skin prick test with undiluted GA, which was negative. We then proceeded to perform the next diagnostic step recommended in the currently available literature: an intradermal test, which was positive at a dilution of 1:100. To rule out a false‐positive reaction, intradermal tests with the same concentrations of GA were carried out in two healthy volunteer persons (SR and GB), who had not previously been exposed to GA. The two subjects also exhibited a positive reaction at the dilution of 1:100. To complete the allergology workup, the CD63 BAT with GA was performed. At the dilution of 1:100, there was a significant activation of the basophilic granulocytes of the patient. To rule out nonspecific basophil degranulation, the CD63 BAT was performed in five more volunteers, including the two individuals who previously had the positive results in the intradermal test. All these persons tested positive in the CD63 BAT with GA at a dilution of 1:100, the same as the patient. At the same time, a skin prick test, an intradermal test, and the CD63 BAT with mannitol, which is a component of injection solutions of GA, were negative in the patient and the two controls.

To identify other similar cases, a search of Medline via PubMed and Google Scholar was conducted using the following search strings: “glatiramer acetate” AND “allergy” or “hypersensitivity” or “sensitization” or “urticaria.” The search was restricted to scientific literature published up to February 2020 in English, German, and French; full texts were systematically retrieved. The bibliographical references of the articles were screened to identify further studies for possible inclusion.

We identified 47 scientific publications. In all, 36 publications were excluded as they were deemed irrelevant. The 11 included publications reported 44 cases of possible immediate hypersensitivity (Table 1). In most cases, positive reactions in the intradermal test occurred at a dilution of 1:100, similarly to our case, or at 1:10.^3^, ^7^, ^9^, ^11^, ^12^, ^13^ In 12 cases, these results were the basis of a diagnosis of type I hypersensitivity.^7^, ^9^, ^11^, ^12^, ^13^ Only some authors tested healthy volunteers^3^, ^5^, ^7^, ^9^, ^11^, ^13^ or performed drug provocation testing in affected patients.^3^, ^9^ In the study of Amsler et al.,^3^ after a series of positive cutaneous reactions, a drug provocation test was performed, in which 16 of the 18 test subjects tolerated GA well. We also recommended drug provocation testing to our patient, who, however, refused it. Our hypothesis was that the patient suffered from chronic spontaneous urticaria that had been initially mistaken for a drug allergy. A similar constellation of conditions was reported by Amsler et al.^3^ in 5 out of 18 patients. Our patient was treated with a fourfold dose of oral fexofenadine 180 mg, as recommended in the national guideline on the treatment of chronic spontaneous urticaria. There was a moderate response to the treatment. The symptoms ceased approximately 26 months after the onset (and 10 months after the discontinuation of the GA therapy). At that time, the patient was being followed up at the neurological clinic and did not require treatment for multiple sclerosis.

**Table 1:** Results of the systematic review: a summary of all available scientific literature on immediate‐type hypersensitivity reactions to glatiramer acetate

Literature/reference	Number of patients	Clinical symptoms	Time to onset of symptoms after injection	Emergency treatment required	Skin prick test (SPT), dilution, and results	Intradermal test (IDT), dilution, and results	In vitro diagnostics	Control persons	Outcome/drug provocation test (DPT)
Amsler E et al., 2017 (3)	18	10/18: urticaria only; 3/8: facial erythema and edema, dizziness; 4/8: chest tightness; 3/8: heat sensations; 2/8: abdominal pain; 2/8: tachycardia or palpitations; 3/8 itching or rash	8/18: immediately to a few minutes; 1/18: 30 min; 5/18: 1 to a few hours; 4/18: unknown	14/18: none; 2/18: emergency consultation, no treatment; 2/18: emergency consultation, corticosteroid with/without antihistamines	16/18: negative with undiluted GA; 2/18: not performed	1/18: positive with undiluted GA; 2/18: positive and 1/18: inconclusive at dilution of 1:10; 7/18: positive and 1/18: inconclusive at dilution of 1:100; 4/18: positive at dilution of 1:1000; 1/18: positive at dilution of 1:10,000; 1/18: negative at all tested dilutions	Not performed	Two controls (who had never received GA) had a positive IDT (at dilution 1:100 or 1:10)	16/18: tolerated DPT without reaction; 1/18: reoccurrence of IPISR; 1/18: not performed
Syrigou E et al., 2015 (4)	1	Injection site redness and swelling followed by generalized urticaria	Unknown	Levocetirizine 5 mg × 4/24 h and corticosteroid therapy (methyl‐prednisolone 60 mg/24 h)	Borderline reaction to undiluted GA	Positive at dilution of 1:10,000	Not performed	Not performed	Successful desensitization and continuation of the drug
Corominas M et al., 2014 (5)	3	Patients 1 and 2: generalized urticaria, nausea, and hypotension; Patient 3: facial angioedema	Patients 1 and 2: upon the first administration of GA (exact time after injection not specified); Patient 3: 20 min after injection	Not reported	3/3 patients tested positive with undiluted GA	Not performed	Specific IgE to GA was determined in the serum of patients and controls using ImmunoCAP Technology, Thermo Fisher Scientific; 3/3 patients had elevated sIgE to GA	10/10 control subjects tested negative in SPT with undiluted GA; 6 patients with MS treated with GA and 10 healthy controls had negative sIgE to GA	Not specified, possibly discontinuation of the drug
Crestani E et al., 2014 (6)	1	Severe shortness of breath, dizziness with shivering, tachycardia, flushing, perioral cyanosis, and brief loss of consciousness	Immediately after injection	By the time of arrival of emergency personnel, the symptoms subsided, and no treatment was needed	Negative with undiluted GA	Positive at dilution of 1:100,000	Not performed	1/1 negative at dilution of 1:100000	Successful desensitization and continuation of the drug
Soriano Gomis V et al., 2012 (7)	3	Patient 1: generalized itching, weals, facial angioedema, dyspnea, and near unconsciousness; Patient 2: facial angioedema, dyspnea, abdominal cramps, and vomiting; Patient 3: generalized itching	Immediately or a few minutes after injection	All patients received epinephrine, dexchlorpheniramine, and methylprednisolone	Patient 1 tested positive in SPT (dilution not specified)	Positive in all three patients at dilution of 1:100	Specific IgE to GA were determined using ELISA, Patients 1 and 2 had elevated levels compared to 10 controls; Patients 2 and 3 tested positive in BAT at 1:100 and 1:400, respectively	3/3 control persons had negative IDT results (dilution not specified); 1/10 controls had elevated sIgE to GA; 1/6 controls had positive BAT result at dilution of 1:20	Not specified, possibly discontinuation of the drug
Baumgartner A et al., 2011 (8)	6	Patient 1: generalized pruritic lesions; Patient 2: generalized cutaneous lesions, hypotension, and dyspnea; Patient 3: generalized erythema, dyspnea, nausea, orthostatic dysregulation, and abnormal fatigue; Patient 4: nausea, facial edema, paresthesia, and bronchial spasm; Patient 5: scalp pruritus, nausea, vomiting, and severe hypotension; Patient 6: heat sensations, facial edema, generalized skin lesions, and bronchial spasm	Immediately to a short time after injection	Patients 1, 2, and 3: none; Patient 4: administration of steroids; Patient 5: administration of dimetindene, dexamethasone, cimetidine, and adrenaline; Patient 6: injection of steroids and clemastine	Not performed	Not performed	Not performed	Not performed	Discontinuation of the drug in all patients
Sánchez‐López J et al., 2010 (9)	3	Patient 1: weals at the injection site and tachycarida; Patient 2: weals at the injection site; Patient 3: weals at injection site, generalized pruritus, and dermographism	Immediately or a few minutes after injection	None	From 1:10,000 to undiluted: negative in all patients	From 1:10,000 to 1:100: positive in patient 1 at 1:10,000; positive in Patient 2 at 1:1000; positive in Patient 3 at 1:100	Sera of patients and controls were examined using ELISA to detect specific IgE to GA; Patient 1 had elevated levels compared to other subjects	5 healthy subjects who had never received the drug and 5 patients with RRMS who tolerated GA: SPT negative in all controls; IDT with dilutions ≤1:100 positive in all controls	Patients 2 and 3 tolerated DPT well, and the treatment with GA was continued; DPT was considered to be too dangerous for Patient 1, therefore, GA was discontinued
Sheth SS et al., 2010 (10)	1	Localized urticaria, nasal congestion, palpitations, diffuse flushing, and hoarseness	Few minutes after injection	Not reported	Undiluted: positive	Not performed	Not performed	1/1 negative in SPT with undiluted GA	Successful desensitization and continuation of the drug
Bains SN et al., 2010 (11)	6	Patients 1 and 2: generalized urticaria without other symptoms; Patient 3: facial swelling, palpitations, throat constriction, and shortness of breath; Patient 4: chest tightness, difficulty breathing, facial flushing, headache, and pruritic skin lesions; Patient 5: development of local hives and swelling at the injection site; Patient 6: local injection site erythema, swelling, and tenderness	Patient 1: 5 h after the first injection; Patient 2: 30 min after injection; Patient 3: immediately after injection; Patient 4: few minutes after injection; Patient 5: few hours after injection; Patient 6: unknown	Patients 1, 3, 5, and 6: none; Patient 2: emergency consultation, administration of epinephrine, diphenhydramine, and a nebulized bronchodilator; Patient 4: oral diphenhydramine	Patient 1: skin testing not performed due to dermographism	Patient 1: skin testing not performed due to dermographism; 3/5 patients positive in IDT with dilution of 1:10 and 2/5 positive in IDT with dilution of 1:100	Not performed	1/9: positive in SPT with undiluted GA, 8/8 positive in IDT with dilution of 1:10 and 6/8 positive in IDT with dilution of 1:100; 4/5 positive in IDT with dilution of 1:1000, and 2/5 positive in IDT with dilution of 1:10,000	Successful desensitization and continuation of the drug
Rauschka H et al., 2005 (12)	1	Generalized flushing, itching urticarial lesions, dyspnea, abdominal cramps, vomiting, and circulatory collapse with unconsciousness for several minutes	2 min after injection	Intravenous (i.v.) adrenalin and antihistamine	Positive with undiluted GA	Positive at dilution of 1:100	Specific IgE was found in serum (laboratory methods were not specified)	Not performed	Discontinuation of the drug
Bayerl C et al., 2000 (13)	1	Nausea, heat sensation, generalized erythema and weals, circulatory collapse, gastric spasms, and diarrhea	Immediately after injection	None	1:10 and undiluted: negative	1:10 and undiluted: both positive after 24 h	Not performed	5/5 control persons were negative in in vivo tests (tests not specified)	Not specified, probably discontinuation of the drug

Abbreviations: GA, glatiramer acetate; IgE, immunoglobulin E; IPISR, immediate post‐injection systemic reaction; MS, multiple sclerosis; RRMS, relapsing‐remitting multiple sclerosis; sIgE, specific immunoglobulin E.

At the present time, there are still no commercially available kits for measuring specific immunoglobulin E (sIgE) antibodies to GA. The presence of these immunoglobulin E (IgE) antibodies can be detected only in specialized laboratories and, therefore, is not a routinely available standardized diagnostic tool. The BAT might be available at a larger number of research facilities, as it is widely used in allergy testing. The diagnostic tools that are widely available are in vivo tests: the skin prick test and the intradermal test. In the report of Amsler et al.,^3^ 13 out of 14 persons who tested positive for GA in the intradermal test tolerated the substance well in the drug provocation test. Thus, false‐positive in vivo test results might not be uncommon. Additionally, in our case, healthy volunteers with no previous exposure to GA tested positive both the in vivo test and the in vitro one. Similar findings have also been reported by other authors.^11^, ^13^ This case highlights that the above‐described symptoms caused by GA may have underlying mechanisms other than IgE‐mediated hypersensitivity, despite the positive allergy test results. Furthermore, the drug does not automatically need to be discontinued based on a positive result in the skin prick test or the intradermal test, as they might be false‐positive due to nonallergic histamine release, which could also explain IPISR. In questionable cases, a drug provocation test appears to be the most reliable of the widely available diagnostic tools at the moment.

## CONFLICT OF INTERESTS

The authors declare that there are no conflict of interests.

## AUTHOR CONTRIBUTIONS

Stefani Röseler treated the patient, performed the in vivo tests, wrote the paper, reviewed, and approved the final manuscript. Fiederike Leufgens performed the systematic literature review, co‐wrote the paper, reviewed, and approved the final manuscript. Hans F. Merk co‐wrote the paper and reviewed and approved the final manuscript. Jens M. Baron co‐wrote the paper and reviewed and approved the final manuscript. Silke Moll‐Slodowy performed the in vitro tests, co‐wrote the paper, reviewed, and approved the final manuscript. Gerda Wurpts co‐wrote the paper and reviewed and approved the final manuscript. Galina Balakirski performed the in vivo tests, performed the systematic literature review, co‐wrote the paper, reviewed, and approved the final manuscript.

## References

[1] Ziemssen T , Schrempf W . Glatiramer acetate: mechanisms of action in multiple sclerosis. Int Rev Neurobiol. 2007;79:537‐570. 17531858 10.1016/S0074-7742(07)79024-4

[2] Ziemssen T , Neuhaus O , Hohlfeld R . Risk–benefit assessment of glatiramer acetate in multiple sclerosis. Drug Saf. 2001;24(13):979‐990. 11735654 10.2165/00002018-200124130-00005

[3] Amsler E , Autegarden JE , Gaouar H , Frances C , Soria A . Management of immediate hypersensitivity reaction to glatiramer acetate. Eur J Dermatol. 2017;27(1):92‐95. 27799134 10.1684/ejd.2016.2907

[4] Syrigou E , Psarros P , Grapsa D , Syrigos K . Successful rapid desensitization to glatiramer acetate in a patient with multiple sclerosis. J Investig Allergol Clin Immunol. 2015;25(3):214‐215. 26182688

[5] Corominas M , Postigo I , Cardona V , Lleonart R , Romero‐Pinel L , Martinez J . IgE‐mediated allergic reactions after the first administration of glatiramer acetate in patients with multiple sclerosis. Int Arch Allergy Immunol. 2014;165:244‐246. 25634237 10.1159/000371418

[6] Crestani E , Lee J , Gorman M , Castells M , Dioun Broyles AF . IgE‐mediated hypersensitivity reaction and desensitization to glatiramer acetate in a pediatric patient. Pediatr Allergy Immunol. 2014;25(8):821‐823. 25200765 10.1111/pai.12267

[7] Soriano Gomis V , Pérez Sempere A , González Delgado P , Sempere JM , Niveiro Hernández E , Marco FM . Glatiramer acetate anaphylaxis: detection of antibodies and basophil activation test. J Investig Allergol Clin Immunol. 2012;22(1):65‐66. 22448457

[8] Baumgartner A , Stich O , Rauer S . Anaphylactic reaction after injection of glatiramer acetate (Copaxone®) in patients with relapsing‐remitting multiple sclerosis. Eur Neurol. 2011;66(6):368‐370. 22123157 10.1159/000334107

[9] Sánchez‐López J , Rodríguez del Rio P , Cases‐Ortega B , Martínez‐Cócera C , Fernández‐Rivas M . Allergy workup in immediate‐type local reactions to glatiramer acetate. J Investig Allergol Clin Immunol. 2010;20(6):521‐523. 21243937

[10] Sheth SS , Posner MA . Modified protocol for desensitization to glatiramer acetate. Ann Allergy Asthma Immunol. 2010;105(2):190. 20674837 10.1016/j.anai.2010.06.003

[11] Bains SN , Hsieh FH , Rensel MR , et al. Glatiramer acetate: successful desensitization for treatment of multiple sclerosis. Ann Allergy Asthma Immunol. 2010;104(4):321‐325. 20408342 10.1016/j.anai.2009.11.040

[12] Rauschka H , Farina C , Sator P , Gudek S , Breier F , Schmidbauer M . Severe anaphylactic reaction to glatiramer acetate with specific IgE. Neurology. 2005;64(8):1481‐1482. 15851756 10.1212/01.WNL.0000158675.01711.58

[13] Bayerl C , Bohland P , Jung EG . Systemic reaction to glatiramer acetate. Contact Dermatitis. 2000;43(1):62‐63. 10902605

